# Aberrant Activation of the Hedgehog Pathway in Cutaneous Melanoma: Therapeutic Potential of Pharmacological Inhibitors

**DOI:** 10.3390/ijms27020762

**Published:** 2026-01-12

**Authors:** Federica Papaccio, Daniela Kovacs, Ramona Marrapodi, Silvia Caputo, Emilia Migliano, Elisa Melucci, Stefano Scalera, Carlo Cota, Marcello Maugeri-Saccà, Barbara Bellei

**Affiliations:** 1 Laboratory of Cutaneous Physiopathology and Integrated Center of Metabolomics Research, San Gallicano Dermatological Institute, IRCCS, Via Elio Chianesi 53, 00144 Rome, Italy; federica.papaccio@ifo.it (F.P.); daniela.kovacs@ifo.it (D.K.); ramona.marrapodi@ifo.it (R.M.);; 2 Department of Plastic and Regenerative Surgery, San Gallicano Dermatological Institute, IRCCS, 00144 Rome, Italy; 3 Department of Pathology, IRCCS Regina Elena National Cancer Institute, 00144 Rome, Italy; 4 Biostatistics, Bioinformatics and Clinical Trial Center, IRCCS Regina Elena National Cancer Institute, 00144 Rome, Italy; 5 Genetic Research, Molecular Biology and Dermatopathology Unit, San Gallicano Dermatological Institute, 00144 Rome, Italy

**Keywords:** melanoma, skin cancer, hedgehog signaling, SMO, sonidegib

## Abstract

Cutaneous melanoma is a highly aggressive skin cancer prone to relapse and metastasis. Surgery is often curative when combined with early screening and prevention. However, in recurrent or advanced disease, the development of new targeted and immune therapies has demonstrated promising clinical outcomes, although the acquisition of resistance limits their effectiveness. Thus, new therapeutic approaches are needed. Emerging data indicate that the Hedgehog (Hh) pathway, which is essential for embryonic development, is aberrantly reactivated in melanoma and may represent a promising therapeutic target. Here, we demonstrate its chronic up-modulation in a panel of patient-derived cell lines and, by investigating the underlying molecular mechanisms, we excluded mutations in the principal components of the pathway. We observed reduced PTCH1 and SUFU repressors expression and GLI2 upregulation as common melanoma features. At the same time, copious SHH release, the principal PTCH1 ligand, evidenced autocrine Hh signaling activation. Consistently, a tendency of greater level of this factor resulted higher in the blood of patients compared to controls, confirming the relevance of ligand-dependent trigger in melanoma. The therapeutic potential of inhibiting the Hh pathway is highlighted by the reduced proliferation and migration observed in the presence of clinically approved pharmacological Hh antagonists. Profiling inflammatory mediators revealed significant modulation upon treatment with SMO inhibitors, possibly affecting chemotactic and immune functions. Collectively, these findings provide deeper insight into the role of the Hh pathway in melanoma and support the potential repurposing of Hh inhibitors as therapeutic agents for melanoma.

## 1. Introduction

Melanoma is the deadliest form of skin cancer, caused by the malignant transformation of melanocytes. Its global incidence is rapidly increasing, posing a significant public health concern [1,2]. The incidence of melanoma is approximately 25 new cases per 100,000 population in Europe, 30 cases per 100,000 population in the USA, and 60 cases per 100,000 population in Australia and New Zealand [2]. Ultraviolet radiation from sunlight is a primary melanoma trigger, causing DNA damage in skin cells and heightening cancer risk. The genetic background also significantly influences individual susceptibility to the disease [3]. Early detection of melanoma lesions in the skin dramatically reduces morbidity and mortality [4]. The primary first-line treatment for melanoma is wide surgical excision. The use of additional therapies depends primarily on the stage and anatomical location of the neoplastic lesion [5]. Despite a wide range of therapeutic options, including targeted therapy (in cases of BRAF-mutated melanoma), immune checkpoint inhibitors, and radiotherapy, drug resistance remains a major clinical challenge [6]. Thus, the development of new drugs and combination therapies is crucial to improving the effectiveness of melanoma treatment. In this context, a comprehensive understanding of the molecular pathways involved in melanoma onset and progression is essential to expanding clinical options.

The evolutionary conserved Hedgehog (Hh) signaling pathway plays a fundamental role during embryogenesis, where it is essential for the morphogenetic specification of tissue patterns [7]. The pathway also plays important roles in adult tissues, including the postnatal physiological regulation of stem cell proliferation and differentiation, as well as the regeneration of adult organs in response to injury [8]. Thus, the easily activatable Hh signaling pathway is considered an adaptive mechanism in tissues frequently exposed to environmental stressors, such as the skin [9]. Hh signaling influences the differentiation of basal cells and helps to maintain skin integrity [9]. It also plays a key role in regulating appendage regeneration, such as hair follicles and sweat glands, which are critical barriers to scarless wound healing [10]. Disruption in this pathway can result in various cutaneous disorders, including hair loss, skin cancer, and developmental abnormalities [10]. In normal development, it is critical for the proliferation and migration of melanoblasts and for maintaining post-natal healthy melanocyte populations in the skin and hair follicles. The Hh signal cascade is essential for the development of ocular melanoblasts in mice and the proper formation of the anterior segment of the eye, as demonstrated by the cell-type-specific activation of Hh signaling via knockdown of Suppressor of Fused (*Sufu*) in the melanocyte lineage in mouse models. *Sufu* ablation affects normal melanoblasts’ migration and homeostasis without impacting their proliferation [11]. However, the same mouse model failed to demonstrate developmental alterations in melanocyte number or distribution within the skin or hair follicles [11]. Moreover, Sonic Hedgehog (SHH) secreted by keratinocytes, in synergy with endothelin-1, has been implicated in nevogenesis and in giant congenital nevi formation [12]. Moreover, murine models with keratinocyte-specific knockdown of Hh components, such as *Ptch1* [13], Smoothened (*Smo*) [14], and *Gli2* [15], demonstrated melanocyte irregularity mostly related to hyperplasia, focally increased pigmentation, and the presence of dendritic pigmented cells in the dermis [15].

In humans, the relationship between Hh signaling and pigmentation is complex. Alopecia is a well-known adverse event associated with the administration of Hh inhibitors, confirming that the Hh pathway plays a role in maintaining hair follicle function [16]. In an in vitro human skin model, inhibition of primary cilia formation activates melanogenesis, opposite to an inducer of this process, inhibiting melanin synthesis underlying the physiological role of ciliogenesis in pigmentation [17]. Additional molecular approaches consisting of SMO agonist supplementation and *GLI2* knockdown also evidenced an inverse correlation between the Hh signal pathway and melanocyte differentiation [18]. Accordingly, a study based on melasma patients proposed that impaired oxidative stress management, as in the case of Nrf2 downregulation, disrupts primary cilia formation, enhances melanogenesis, melanosome transfer and keratinocyte differentiation, ultimately resulting in skin hyperpigmentation [19].

More importantly, it has become increasingly clear that the Hh pathway is associated with higher cancer prevalence, malignant progression, and poor prognosis [20]. Germline mutations in constituents of the Hh pathway have been linked to an increased cancer risk; for example, mutations in these components, as seen in Gorlin-Goltz Syndrome, predispose individuals to basal cell carcinoma (BCC), medulloblastoma, and rhabdomyoma [21]. Somatic inactivating mutations of PTCH1 have been identified in 70–90% of BCCs, placing Hh signaling among the driver pathways in skin BCCs [22]. Notably, BCCs are sometimes pigmented with melanocytes unusually located around the edges or within the lesion, clinically resembling melanoma [23]. Moreover, mutations in *PTCH1* have been frequently reported in other malignancies, including lung, colorectal, and stomach cancers [24], as well as breast cancer [25].

Aberrant activation of the Hh signaling pathway can arise through both ligand-dependent and ligand-independent mechanisms. Ligand-independent activation results from mutations in various components of the Hh pathway, which mimic the effects of ligand stimulation [26]. Conversely, in the ligand-dependent mode, chronic pathway activation is sustained by autocrine and paracrine production of Hh ligands by transformed tumor cells and/or the surrounding stroma.

In mammals, these ligands comprise SHH, Indian Hedgehog (IHH), and Desert Hedgehog (DHH). Ligand engagement with the transmembrane receptors PTCH1, PTCH2 alleviates their inhibitory effect on SMO, facilitating its accumulation within the primary cilium, thereby initiating downstream signal transduction [27]. SMO mediates downstream signaling by dissociating GLI proteins from Kif7 and SUFU. Activated GLI transcription factors (GLI1, GLI2, GLI3) accumulate in the nucleus and regulate Hh target genes. GLI1 and GLI2 promote G1/S and G2/M cell cycle phase progression, driving the transition from quiescence to proliferation [28]. In contrast, GLI3 functions primarily as a transcriptional repressor of Hh signaling during normal development, owing to its proteolytic processing into a truncated repressor form. Upon Hh pathway activation, particularly through Shh, the cleavage of GLI3 is attenuated. In this context, GLI3 can function as a positive regulator by directly targeting the GLI1 promoter and promoting its expression. This mechanism highlights GLI3’s dual role depending on its processing state within the pathway [29]. In line with the critical role of Hh signaling in tumor biology, numerous molecules have been developed over the years to target this pathway. Vismodegib (GDC-0449; trade name Erivedge) and sonidegib (LDE-225; trade name Odomzo), both SMO inhibitors, have been approved for oral treatment of patients with BCC or those with recurrent, locally advanced BCC who are not suitable candidates for surgery or radiotherapy.

Recently, the involvement of the Hh signaling pathway in melanoma initiation has been increasingly suggested [30,31,32,33,34]. Moreover, to date, evidence supporting the clinical efficacy of Hh inhibitors in melanoma remains lacking, particularly data derived from a more reliable model, such as patient-derived samples.

This study aimed to evaluate the possible use of Hh inhibitors for melanoma therapy by dissecting the activity of the pathway and investigating the biological effects of commercially available pharmacological inhibitors on a panel of patient-derived melanoma cells.

## 2. Results

### 2.1. The Hh Signaling Pathway Is Overactive in Human Melanoma Cells

Compared to commercially available tumor cell lines, fresh patient-derived cells retain key features of tumor heterogeneity, enabling better assessment of drug responses. We previously demonstrated that primary low-passage melanoma cells mirror the corresponding in vivo phenotype [35]. Here, we used a panel of 19 patient-derived melanoma cell lines, including both primary and metastatic samples, to assess Hh pathway activity in comparison to normal human melanocytes (NHMs, *n* = 15) isolated from clinically healthy skin of unaffected individuals. Patient and control specimen characteristics are reported in the Supplementary Materials (Tables S1 and S2).

**Figure 1:**
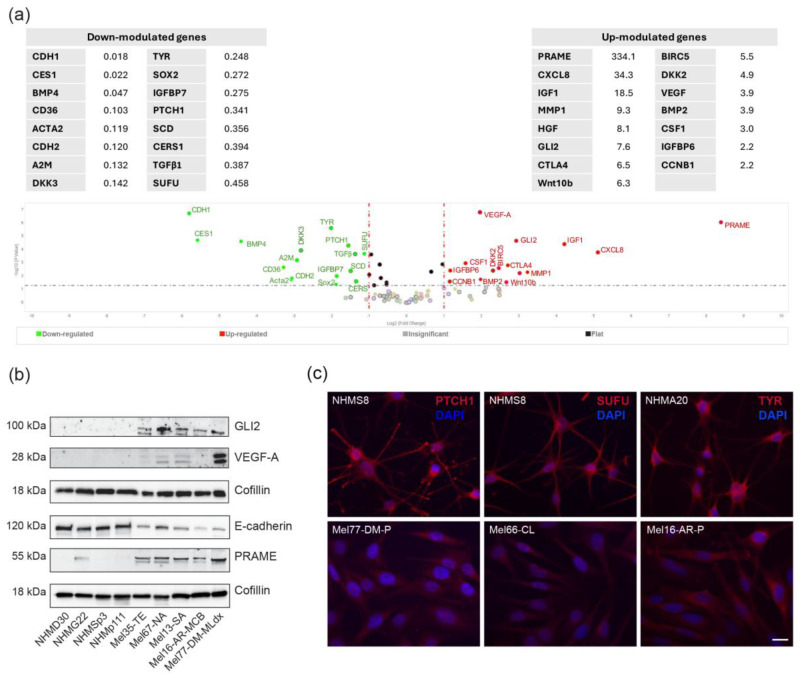
Comparative analysis of Hh pathway-related gene and protein expression in melanoma cells and NHMs. (**a**) Volcano plot showing the overall analysis of gene expression. Melanoma cells (biogroup 1, *n* = 19) were compared to NHMs (biogroup 2, *n* = 15), arbitrarily defined = 1. Tables show the values of significantly down- (left panel) and up- (right panel) regulated genes. A one-way ANOVA statistical test was performed with the following thresholds: significant increase: >2.0-fold change and *p* < 0.05 (marked in red); significant decrease: <0.5-fold change and *p* < 0.05 (marked in green); any fold difference with *p* ≥ 0.5, e.g., insignificant (marked in gray); ≤2.0-fold change or ≥0.5-fold change, e.g., flat, (marked in black). (**b**) One representative Western blot analysis for GLI2, VEGF-A, E-cadherin and PRAME expression in NHMs (*n* = 8) and melanoma cells (*n* = 10) (**c**) One representative immunofluorescence analysis of PTCH1, SUFU and Tyrosinase (TYR) expression in NHMs (*n* = 3) and melanoma cells (*n* = 8). Nuclei are counterstained with DAPI. Scale bar: 20 µm.

First, we used a gene expression array card system to evaluate a total of 90 genes of interest related to the Hh pathway, Wnt pathway, pigmentation, and pro-tumoral factors (listed in Table S3). Clustering the samples into two biogroups (biogroup 1: melanoma vs. biogroup 2: NHMs, arbitrarily defined as 1), we identified 16 significantly downregulated genes and 15 significantly upregulated genes (Figure 1a).

The mRNA coding for *GLI2*, one of the most important downstream effectors of Hh signaling, was markedly upregulated (7.59-fold increase) in melanoma cells, whereas two key Hh repressors, PTCH1 and SUFU, were significantly reduced (0.341 and 0.458-fold decrease, respectively) compared to controls. These data, further supported by confirmation at the protein level (Figure 1b), suggest that the components necessary for active Hh signal cascade are present in melanoma cells.

The most relevant transcriptomic data included the overexpression of PReferentially Expressed Antigen in MElanoma (*PRAME*), a tumor-associated antigen used for the differential diagnosis of melanocytic tumors [36], C-X-C Motif Chemokine Ligand 8/Interleukin-8 (*CXCL8/IL-8*), a chemokine that promotes melanoma cell proliferation, survival, migration, and invasion by interacting with CXC chemokine receptor type 1 (CXCR1) and CXC chemokine receptor type 2 (CXCR2), insulin-like growth factor (*IGF-1*) as well as its interactor insulin-like growth factor binding protein 6 (*IGFBP6)*, both implicated in melanoma onset and epithelial–mesenchymal transition process [37]. Significantly upmodulated genes also included Hepatocyte Growth Factor (*HGF*), having a significant role in the progression and metastasis of melanoma by promoting tumor cell survival, proliferation, and migration [38,39], Cytotoxic T-Lymphocyte Associated Protein 4 (*CTLA4*), Wingless-Type MMTV Integration Site Family, Member 10B (*WNT10b*), Baculoviral IAP Repeat Containing 5 (also known as Survivin *BIRC5*), Dickkopf WNT Signaling Pathway Inhibitor 2 (*DKK2*), Vascular Endothelial Growth Factor (*VEGF*), Bone Morphogenetic Protein 2 (*BMP2*), Colony Stimulating Factor 1 (*CSF1*) and Cyclin B1 (*CCNB1*). Opposite, the transcript for *CDH1*, gene coding for the adhesion molecule E-cadherin, *CES1* and *BMP4* appeared the most down-modulated genes. An interesting antitumorigenic activity has been demonstrated for BMP4 protein, since it reduces VEGF expression, thereby inhibiting angiogenesis within the tumor microenvironment and consequently suppressing tumor growth [40]. Correspondingly, the transcript coding for VEGF was higher in melanoma cells compared to controls. The biological significance of reduced CES1 is less supported by the literature since inhibition of CES1 in vitro and in vivo has been associated with diminished melanoma progression [41]. In line with the gene expression and protein levels of E-cadherin, arguing for mesenchymal-like/undifferentiated phenotype, the amount of tyrosinase mRNA resulted significantly lower, confirming a less differentiated feature of tumor cells when compared to epithelial melanocytes. Of interest, the gene expression of β2-Microglobulin (β2), a major subunit of major histocompatibility complex (MHC) class I, with important biological functions and roles in melanoma immune response [42], was significantly lower compared to NHMs. Transcriptomic data verification was performed by sampling, not on the full data set, combining Western blot and immunofluorescence analyses (Figure 1b).

**Figure 2:**
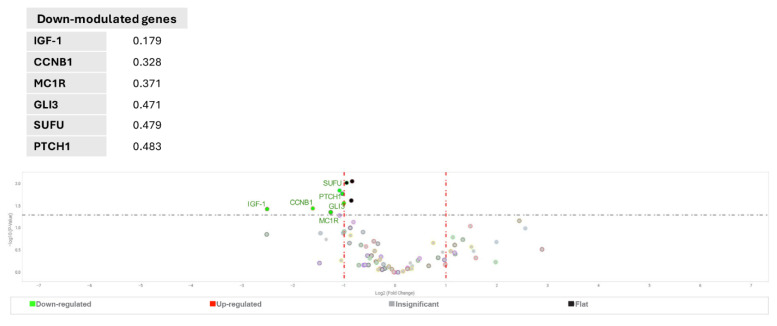
Hh pathway activity in primary and metastatic melanoma cells. Volcano plot showing the analysis of gene expression in primary (*n* = 12) and metastatic (*n* = 7) melanoma cells, with the corresponding table showing the values of significantly down-regulated genes. A one-way ANOVA statistical test was performed with the following thresholds: significant increase: >2.0-fold change and *p* < 0.05 (marked in red); significant decrease: <0.5-fold change and *p* < 0.05 (marked in green); any fold difference with *p* ≥ 0.5, e.g., insignificant (marked in gray); ≤2.0-fold change or ≥0.5-fold change, e.g., flat, (marked in black).

Separated analysis of primary and metastatic samples revealed significantly lower expression of *PTCH1* and *SUFU* in advanced melanoma, confirming the relevance of the Hh signaling pathway in melanoma progression (Figure 2).

Interestingly, a lower level of GLI3, which predominantly serves as a repressor of Hh pathway target genes, emerged as a distinctive marker for metastatic melanoma compared to cells derived from primary lesions. In line with this, a 2.5-fold increase in GLI1 expression was observed in metastatic melanoma, although this difference did not achieve statistical significance. Furthermore, comparative analysis between cell lines harboring *BRAF* mutation (*n* = 8) and *NRAS* mutations (*n* = 6) versus wild-type genotype (*n* = 5) revealed a significantly elevated level of GLI2 in the first group, with a fold change of 3.702, *p* = 0.0276, and 5.264, *p* = 0.00118, respectively, confirming previous data that associated abnormal function of Hh signaling activation with MAPKs pathway chronic activation [43]. Collectively, the screening of patient-derived melanoma cells evidenced that switching on Hh signaling is part of the melanomagenesis process.

### 2.2. Hedgehog Pathway Mutational Profile in Melanoma Cells

Somatic mutations disarrange Hh signaling in numerous sporadic solid tumors, including BCC, medulloblastoma, rhabdomyosarcoma and T-cell acute lymphoblastic leukemia [44,45,46,47]. Reasoning that Hh mutational status might also be implicated in melanoma, we evaluated ten key Hh pathway genes by DNA next-generation sequencing (NGS) analysis in 23 cell lines derived from 17 patients. Specifically, 16 primary tumors and 7 metastases (4 cutaneous and 3 lymphonodal) were analyzed with a custom-designed gene panel (Table S4) [48]. All 23 melanoma cell lines evaluated resulted in wild-type configuration for the ligands *SHH*, *DHH*, and *IHH*, the repressors *PTCH1*, *PTCH2*, and *SUFU,* the effectors *SMO*, *GLI1*, *GLI2*, and *GLI3* genes, indicating that deregulation of the Hh intracellular signaling might arise from mechanisms not involving mutational events.

### 2.3. SMO Antagonists Reduce the Proliferative Capacity of Melanoma Cells and Induce Apoptosis In Vitro

**Figure 3:**
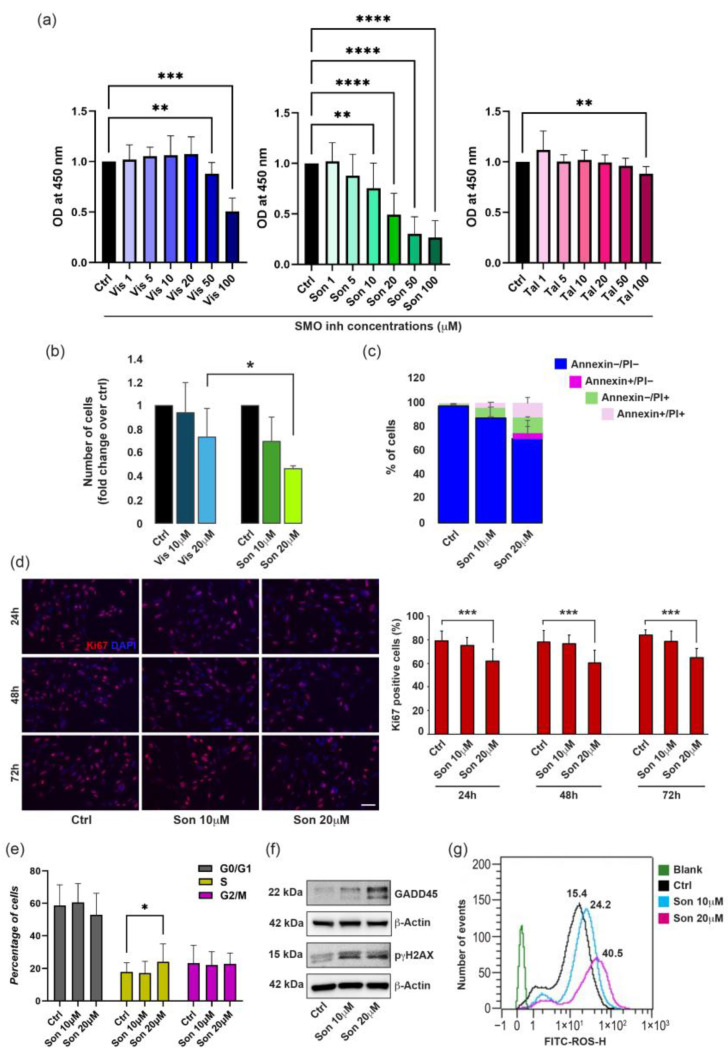
Effects of SMO inhibitors on melanoma cell viability, growth, and apoptosis. (**a**) MTT assay of melanoma cells (*n* = 13) treated in duplicate with increasing concentrations (range 1–100 µM) of vismodegib (Vis), sonidegib (Son) and taladegib (Tal) for 72 h. Results are expressed as fold change ± SD relative to control (Ctrl) cells. (**b**) Cell count of vismodegib- and sonidegib-treated cells in comparison to untreated control samples. Data are representative of six different experiments performed in duplicate. (**c**) Annexin/PI staining evidenced a dose-dependent increase in the percentage of apoptotic cells under the effect of sonidegib. Histograms are representative of three different cell lines examined in duplicate. (**d**) One representative immunofluorescence staining for Ki67 in sonidegib-treated and control cells (*n* = 11) and quantitative analysis of the number of positive Ki67 cells in response to Son treatment (10–20 µM) at 24, 48 and 72 h. Results are expressed as the mean value of positive cells/total cells (%). Nuclei are counterstained with DAPI. Scale bar: 50 µm. (**e**) Cell cycle distribution evaluated by flow cytometric analysis on melanoma cells treated with sonidegib 20 µM for 72 h. (**f**) One representative Western blot reporting the increased expression of GADD45 and pγH2AX in the presence of sonidegib. (**g**) One representative overlay of the median fluorescence intensity (MFI) of stained negative control cells and samples treated or not with sonidegib at the concentration of 10–20 µM. * *p* < 0.05; ** *p* < 0.01; *** *p* < 0.005; **** *p* < 0.001.

To evaluate the therapeutic potential of Hh pathway antagonists, we selected two molecules already approved for BCC treatment: vismodegib (GDC0449; trade name Erivedge) and sonidegib (LDE225; trade name Odomzo) and taladegib (LY2940680), which is presently under consideration in several clinical phase I/II tumor studies (documented in http://clinicaltrials.gov at the date of 29 December 2025). All these compounds block Hh signal transduction through direct interaction with SMO [47]. First, we performed a preliminary set of experiments using a range of concentrations from 1 µM to 100 µM for 24, 48 and 72 h of continuous treatment before performing an MTT assay. Data reported in Figure S1 show a time-dependent decrease in metabolic activity, evidencing a stronger effect in the case of sonidegib. Since maximum response was observed with the longer treatment, we adopted this endpoint to further evaluate drugs response in a wide panel of melanoma cell cultures. Extending the analysis on a total of 13 different cell lines (Figure 3a), we demonstrated a robust dose-dependent cytotoxic effect exerted by sonidegib, whereas vismodegib and taladegib reached a significant reduction in cell viability only at the higher doses.

Separated analysis of *BRAF*/*NRAS*-mutated melanoma cells and those harboring wild-type configuration of these genes revealed no significant differences in SMO inhibitor toxicity (Figure S2a). Similarly, comparative analysis between primary and metastatic cell lines yielded comparable results (Figure S2b). Differences in cytotoxicity between sonidegib and vismodegib were additionally proved by cell count (Figure 3b), consistent with our previous observation [49]. Additionally, Annexin/PI staining demonstrated that the apoptotic process underlies the observed cell death (Figure 3c). Based on its efficacy, sonidegib was selected at doses of 10 and 20 µM for further investigation. We evaluated its impact on cell growth by analyzing the proliferation marker Ki67 via immunofluorescence in a panel of primary and metastatic melanoma cells treated with the Hh inhibitor for 24, 48, and 72 h. Sonidegib at 10 µM showed a trend toward reducing the number of Ki67-positive cells compared to vehicle. At 20 µM, the proportion of cell lines exhibiting growth inhibition increased, with significant reductions at 24, 48, and 72 h (Figure 3d). Taken together, the minor expression of the Ki67 proliferation marker and the mild decrease in metabolic activity recorded by MTT assay at early time points (24 and 48 h) suggest an initial cytostatic effect, which resulted in significant cell death only after prolonged drug treatment.

**Figure 4:**
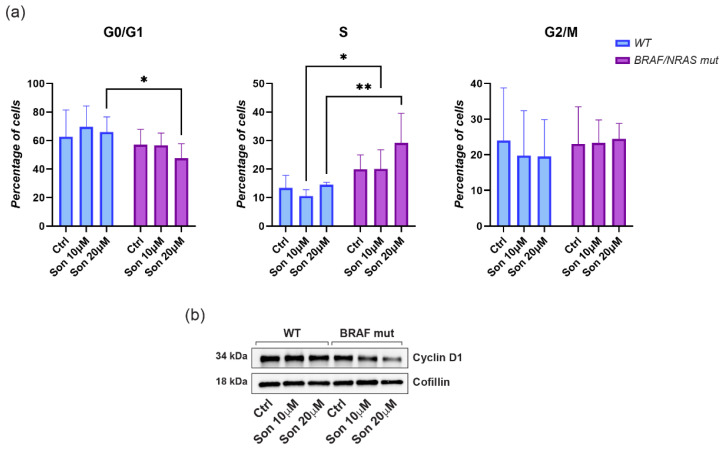
Comparative effects of sonidegib on cell cycle phases distribution in wild-type and *BRAF*/*NRAS* mutated melanoma cells. (**a**) The analysis of distribution in different phases of the cell cycle was analyzed grouping cells according to the genotype for *BRAF*/*NRAS* genes. Mutant cells (*n* = 6) showed a higher baseline proportion of S-phase cells compared to wild-type cells (*n* = 2) and an enhanced increase in S-phase entry upon treatment. At the same time, a significant reduced percentage of cells in G0/G1 characterized mutated cells with respect to wild-type samples. No significant differences are observed in the G2/M phase in either genotype. (**b**) Immunoblot of Cyclin D1 expression of one representative WT and one representative *BRAF*-mutant cell line following treatment with sonidegib or not (untreated control cells). Cofilin was used for loading control. * *p* < 0.05; ** *p* < 0.01.

To better characterize sonidegib-dependent effects on cell proliferation, we analyzed the cell cycle after treatment with the two concentrations of the inhibitor. Melanoma cells were synchronized at the G0/G1 phase via serum starvation. Cell cycle progression was then analyzed at 24, 48, and 72 h after treatment with either sonidegib or DMSO (vehicle control). Notably, treatment with 20 µM led to an accumulation of melanoma cells in the S phase of the cell cycle, accompanied by a corresponding decrease in the G0/G1 population compared to the control. The effect was consistently observed across all analyzed time points, with a marked increase at 72 h (Figure 3e). S-phase arrest is indicative of the accumulation of a multinucleated cell population, possibly due to DNA damage and impaired completion of DNA replication, which prevents progression to the subsequent cell cycle phase. Consistently, sonidegib treatment increase the level of Growth arrest and DNA damage-inducible protein (GADD45) and pγH2AX, two factors implicated in cell cycle arrest and DNA repair (Figure 3f). Supporting the idea that sonidegib causes a replication stress, treated cells showed an early dose-dependent increase in intracellular reactive oxygen species (ROS), indicative of oxidative stress (Figure 3g). When melanoma cells are grouped according to their genotype (WT vs. BRAF/NRAS-mutated), treatment with sonidegib results in a significant dose-dependent enhancement in the percentage of cells in the S phase only in mutant cells. In parallel, BRAF/NRAS-mutated cells, but not those carrying the wild-type genotype, showed a decrease in the proportion in the G0/G1 phase compared to untreated samples, which was accompanied by a decrease in the CyclinD1 protein expression (Figure 4a,b).

Activated RAS/MAPK signaling has been shown to enhance Hh pathway activity by promoting the nuclear translocation and transcriptional activation of GLI1 and GLI2. This crosstalk amplifies Hh signaling downstream of canonical components, contributing to increased pathway activity independently of SMO regulation [33]. Consistently, in melanoma cell lines carrying BRAF or NRAS mutation, GLI2 expression was stronger than in wild-type cells. Thus, a combination of GLI and MAPKs inhibitors may result in an enhanced therapeutic effect than single agents in treating melanoma.

### 2.4. Inhibition of SMO Stimulates the Autocrine Production of Hh Ligands

**Figure 5:**
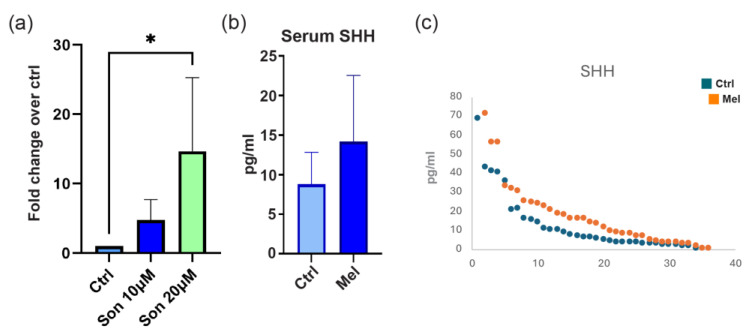
SHH production in response to sonidegib treatment. (**a**) Soluble SHH quantitation in the culture medium by ELISA on 11 melanoma cell lines treated with sonidegib at 10 µM and 20 µM for 48 h. Data reported fold-change relative to untreated controls. (**b**) ELISA quantification of circulating SHH assaying the serum of melanoma patients (*n* = 58) and healthy subjects (*n* = 50). Histograms report mean values with 95% confidence intervals (CI). (**c**) Distribution of individual value of serum concentration of SHH. * *p* < 0.05.

Since the lack of mutation in the Hh pathway ruled out a ligand-independent mechanism, we tested the ligand-dependent route to explain the abnormal Hh activity. For this purpose, we measured the concentration of SHH, the principal Hh family ligand factor. Immunoenzymatic analysis of soluble SHH in the cell culture medium evidenced an intense production of this factor by melanoma cells, proving the autocrine mechanism for the observed activation of the corresponding signaling (Figure 5a).

Moreover, the concentration of SHH dose-dependently augmented in the presence of the SMO antagonist, arguing for an intrinsic tight modulation of the signaling by melanoma cells and the endeavor to counteract its suppression imposed by pharmacological treatment. This observation evidences a pivotal role of Hh in melanoma biology.

To confirm the clinical relevance of ligand-dependent mechanism of Hh cascade activation in melanoma, we performed immunoenzymatic quantification of the Shh ligand in the serum of a panel of 58 melanoma patients compared to healthy control subjects (*n* = 50). Data reported in Figure 4b revealed an overall higher level of circulating ligand in the melanoma group, although the difference did not reach statistical significance, possibly due to the small sample size.

### 2.5. SMO Inhibition Counteracts Melanoma Cell Migration Impacting the Expression of Adhesion Molecules

**Figure 6:**
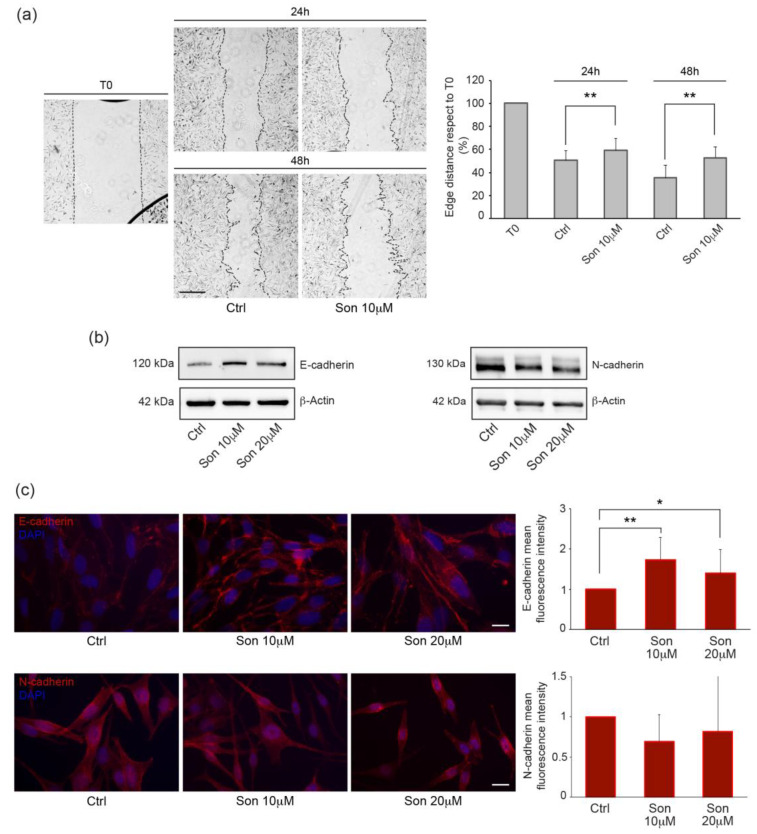
Effects of sonidegib on melanoma cell migration and adhesion protein expression. (**a**) Representative images of a wound scratch assay and corresponding quantitative analysis of the distance between the wound edges performed on melanoma cells (*n* = 11) treated with 10 µM sonidegib for 24 and 48 h. Results are shown as fold reduction compared to the T0 value, which was set at 100. Scale bar: 100 µm. (**b**) Representative Western blots for E-cadherin and N-cadherin expression in melanoma cells treated with 10 and 20 µM Son for 48 h. (**c**) Representative immunofluorescence analysis and corresponding signal intensity measurement of E-cadherin and N-cadherin in melanoma cells treated with 10 and 20 µM Son for 48 h. Nuclei are counterstained with DAPI. Scale bar: 20 µm. The results are presented as fold changes relative to the mean fluorescence intensity per cell in vehicle-treated controls, which were normalized to a value of one. * *p* < 0.05; ** *p* < 0.01.

Previous studies reported a possible anti-migratory activity of SMO inhibitors on melanoma cells [31,33,50,51]. Thus, we interrogated our patient-derived cell lines, performing the wound scratch assay in the presence or absence of the compound. Measurement of the distance between the scratch edges by image analysis showed a progressive decrease in both treated and untreated cells at 24 and 48 h, juxtaposed to immediately after the scratch (T0). The comparison between sonidegib-treated cells and vehicle-treated cells showed a significantly reduced ability of the former to recover the scratched area in 72.7% of the cell lines at 24 h, which became statistically significant for all the samples evaluated at 48 h (Figure 6a).

The migratory and metastatic potential of melanoma cells has been linked to changes in the expression of cadherins, a family of adhesion molecules [52,53]. A progressive decrease in E-cadherin levels is an early sign of melanoma progression, leading to a gradual loss of interactions between cancer cells and surrounding keratinocytes. A parallel increase in N-cadherin on the cell surface often occurs, allowing melanoma cells to interact with fibroblasts, thereby promoting deeper invasion. Hence, based on the results of the scratch assay, we investigated the effect of sonidegib on cadherin expression levels. Western blot analysis evidenced augmented E-cadherin expression in sonidegib-treated cells (Figure 6b). Image analysis, considering the quantification and localization of the fluorescence signal, revealed an overall increase in E-cadherin expression, proving the correct transmembrane pattern in most cells constitutively expressing this protein (Figure 6c). However, basal expression of this adhesion protein widely varied among samples (Figure S3a) and E-cadherin was undetectable in some cell lines analyzed. In these cases, treatment with sonidegib failed to re-induce its expression (Figure S3b). In parallel, analysis of N-cadherin showed no significant changes in its expression levels (Figure 6b,c).

Confirming the link between the metastatic potential and the activation of the Hh signaling pathway, we observed a tight correlation connecting GLI2 and N-cadherin expression (Figure S4a). Moreover, clustering samples for wild-type, *BRAF* and *NRAS* genotypes, a clear association between MAPK signaling and Hh pathway, and pro-migratory feature was evident (Figure S4b). Notably, NRAS mutated cells showed the highest levels of GLI2 and N-cadherin expression.

### 2.6. SMO Inhibition Impact on the Production of Inflammatory Mediators

Chronic inflammation promotes phenotypic plasticity in melanoma cells, facilitating their adaptation and survival under therapeutic pressure. Inflammatory signaling within the tumor and proximal microenvironment significantly influences the efficacy of targeted therapies and immune checkpoint inhibitors [54]. In this study, we employed high-throughput proteomic arrays, including 58 proteins involved in immunity, to assess the impact of SMO inhibition on key inflammatory mediators in melanoma cells.

For this purpose, conditioned media were collected from 16 melanoma cell lines (11 primary and 5 metastatic) after 48 h of treatment with 20 μM sonidegib. Globally, treatment induced a complex and heterogeneous modulation of immune mediators, predominantly promoting enhanced inflammation (Table 1).

Modifications showing at least a ≤0.5-fold decrease or a ≥2.0-fold increase and/or statistical significance (*p* ≤ 0.05, any change) are reported in Table 2. All other statistically non-relevant results are provided in Table S5. Among the analyzed interleukins, IL-1α, IL-1β, IL-4, IL-5, IL-9, and IL-10 demonstrated an upward trend, with IL-4 reaching statistical significance. The intensification of IL-4 expression, even if modest, is of interest since IL-4 is one of the most potent regulators of type 2 inflammation. However, IL-4 demonstrated an antiproliferative activity that can be enhanced in combination with IFN-γ or TNF-α [55]. In preclinical models, IL-4 overexpression can rescue T cell immune functions, improving tumor control [56]. Stimulation with IL-4 enhances the NK cell cytotoxicity against metastatic melanoma patients [57]. Accordingly, it has been proposed in immunotherapies to boost immune responses against both mouse and human tumors [58]. Conversely, other studies have implicated IL-4 in promoting pro-tumoral microenvironment remodeling [59] and resistance to checkpoint inhibitors, because IL-4 exerts detrimental effects on immune cells, compromising the differentiation of Th1 cells [60]. Opposite, sonibegib significantly reduced the release of LIGHT (also referred to as tumor necrosis factor superfamily member 14, TNFSF14). When LIGHT molecules are delivered from tumors, they induce significant changes in the microenvironment, primarily through vascular normalization and the formation of tertiary lymphoid structures. These changes can synergize with treatments that enhance anti-tumor immune responses, such as checkpoint inhibitors or cancer vaccines, significantly improving the effectiveness of immunotherapy against cancer [61]. The modulation of LIGHT expression may have dual effects on T-cell-mediated immunity: when released by melanoma cells, this TNF family member can induce a proliferative response in CD3 + CD8 + T cells; however, it also plays a role in co-stimulating the apoptotic response of these cells, facilitating tumor suppression [62]. Moreover, LIGHT is one component of the risk signature in glioblastoma [63], a type of tumor deeply influenced by Hh signal pathway.

Another class of secreted proteins implicated in immune system recruitment that consistently shows reduced levels with SMO inhibition includes the monocyte/macrophage chemoattractant MCP factors (MCP-2, MCP-3, MCP-4), macrophage-derived chemokine (MDC), and macrophage-stimulating protein (MSP), often resulting in undetectable levels post-treatment. Specifically, these secreted factors were detectable at basal levels in some, but not all, cell lines. However, sonidegib exposure induced a significant change (*p* < 0.0001), as they completely disappeared from the conditioned medium. An important oncogenic role has been attributed to MCP-2 since it fosters the local environment into a prometastatic niche [64]. MDC (macrophage-derived chemokine, also known as CCL22) gene expression is increased in early-stage melanoma [65]. MDC plays a role in attracting T regulatory cells (Treg), which can suppress the immune response against cancer cells. However, increased MDC expression can also correlate with immunosuppression, highlighting the complex interplay of immune responses in melanoma. Considering that several microenvironmental factors regulate the impact of MDC, the prediction of its expression appears too speculative.

Notably, granulocyte chemotactic protein 2 (GCP-2) exhibited a significant increase relative to untreated samples, implicating enhanced neutrophil-associated inflammatory signaling.

The increment of SDF1a, also known as C-X-C motif chemokine ligand 12 (CXCL12), might represent an undesired effect since the interaction with its receptor CXCR4 sustains cell proliferation, invasion, and survival via downstream signaling pathways [66].

Other immune modulators, such as lymphotactin (TSLP) and macrophage migration inhibitory factor (MIF), MIP-1α and β, Rantes (CCL5) and TNFα showed heterogeneous and non-significant increase. MIP-1α (Macrophage Inflammatory Protein 1-alpha), also known as CCL3, is a chemokine that attracts immune cells like T cells, macrophages, and dendritic cells to the tumor, influencing the immune response against melanoma [67]. The subcutaneous administration of these cytokines notably increased the expression of several other chemokines and led to a reduction in primary melanoma growth in mice lacking MCP-1 or MIP-1α. They also suppressed primary tumor development in wild-type mice. These findings suggest that MCP-1 and MIP-1α play a crucial role in mediating protective anti-tumor immune responses against melanoma, primarily by enhancing lymphocyte infiltration into the tumor and stimulating subsequent cytokine production [68]. Very recently, clinical analysis revealed that high Rantes expression is linked to improved survival outcomes and a more favorable response to anti-PD1 treatment in melanoma patients, potentially through the induction of M1 macrophage polarization [69]. Differently, an intense expression of Rantes has been linked to tumor aggressiveness, and experiments have demonstrated that this protein favors tumor progression [70].

### 2.7. SMO Inhibition Modestly Affects the Production of Growth Factors by Melanoma Cells

Extending the analysis to growth factors with potential autocrine and paracrine functions, we observed a generally mild effect. Among the 40 proteins analyzed, 21 resulted undetectable or barely detectable in most of the tested samples and where not considered for statistical analysis, whereas 16 showed a modest variation with no statistical significance. Only three molecules were significantly modulated by sonidegib: growth differentiation factor 15 (GDF-15), insulin, and osteoprotegerin (OPG) (Table 2). All other statistically non-relevant results are provided in Table S6.

From a broader perspective, the elevation of pro-mitogenic molecules represents a potential “adverse” effect, possibly linked to the observed cell cycle dysfunction. Notably, GDF-15, also known as macrophage inhibitory cytokine-1, is overexpressed in melanoma cells in vitro compared to normal human melanocytes, and higher levels of this factor in vivo are associated with metastatic disease [71]. Moreover, GDF-15, a cytokine that is abundantly produced by many cancer types, was shown to interfere with T cell activation and anti-PD-1-mediated checkpoint inhibition [72]. Consistently, the circulating level of GDF-15 in the blood of melanoma patients has been proposed to predict anti-PD-1 therapy outcome [72]. Autocrine production of GDF-15 has also been implicated in tumor vascularization during melanoma development in an in vivo mouse model [73]. On the other hand, GDF-15 is a stress-responsive cytokine involved in mitochondrial homeostasis and sometimes referred to as “mitokine” [74]. GDF-15 integrates the response of metabolic stress and DNA damage [75,76], suggesting that its stimulation may represent an attempt to overcome replicative stress associated with the cell cycle block imposed by the inhibition of the Hh pathway.

OPG (osteoprotegerin or TNFRSF11B) physiologically regulates bone metabolism and angiogenesis. However, the production of its soluble form has been reported in metastatic melanoma [77,78], but an explanation of its role in melanoma biology is still lacking. The significant rise in the insulin released by melanoma cells might be a metabolic consequence of the oxidative stress evidenced by ROS elevation and possibly impacting metabolic function. There is poor information in the literature regarding insulin autocrine/paracrine function in melanoma; however, a study by do Prado and collaborators not long ago reported preliminary data concerning the inhibition of melanoma growth in mice through the insulin-induced expression of activating transcription factor 4 (ATF4) [79].

## 3. Discussion

Drug repositioning efficiently cuts the time cost, investments, and risks of the early stage of drug discovery. Sonidegib and vismodegib are successfully used as targeted treatment for advanced or metastatic BCC, and their repurposing in oncology is attractive. Both molecules function as antagonists for SMO and lead downstream transcription factors GLI1 and GLI2 to remain inactive.

Stimulation of Hh signaling sustains the proliferation of normal human melanocytes in vitro, and sporadic previous studies on commercially available melanoma cell lines, as well as histochemical investigations, provided preliminary evidence for the involvement of the Hh signaling pathway in the occurrence and development of melanoma [49,80,81]. Additional corroboration for targeting Hh emerged from the computational approach (in silico target fishing) and derived in vitro evaluation [82]. By integrating all this information, we selected sonidegib, vismodegib and taladegib as candidate drugs for melanoma therapy, and we interrogated our patient-derived melanoma cell lines collection to support the possible clinical treatment of melanoma with Hh inhibitors. First, we provided evidence that melanoma cells extracted from fresh biopsies have Hh signaling chronically activated. Gene expression profiling revealed downregulation of two repressors, PTCH1 and SUFU, along with upregulation of the downstream effector GLI2 in melanoma cells compared to normal melanocytes. Notably, cells derived from advanced metastatic lesions exhibited a more pronounced phenotype, characterized by significantly lower levels of three key repressor proteins, PTCH1, SUFU, and GLI3, compared to primary melanoma cells. Contrarily, a previous study reported abnormally elevated PTCH1 levels in WM278, WM3248, A375, and SKMel94 melanoma cell lines [31]. The difference may lie in the mechanism responsible for Hh pathway activation, since in our cell lines SHH is abundantly produced and, in the presence of this ligand, the engagement of PTCH1 in primary cilia triggers its internalization and a degradative feedback loop that limits signal output in response to the ligand [83]. By contrast, in the absence of SHH, PTCH1 stably binds to SMO, possibly affecting the protein’s steady-state level.

In this study, mutational analysis together with immunoenzymatic quantification of SHH argues for a ligand-dependent autocrine mechanism as the prevalent way for the activation of the Hh cascade in melanoma. Notably, in vitro neutralizing antibody against Hh ligands blocked the growth stimulating capacity of these factors, furtherly supporting the relevance of ligand-dependent autocrine mechanism in cancer [84,85]. Hh ligands released by tumor cells also influence the surrounding stroma, which, in turn, provides a more favorable environment for tumor growth [86] including melanoma [87,88,89]. Abnormal Hh signaling pathway plays a role in immune evasion in various cancers by modulating tumor immune/stroma cell phenotype and function, such as macrophage polarity, T cell response, and fibroblast activation, as well as their mutual interactions between tumor cells and nonneoplastic cells [90]. Accordingly, among the genes investigated, *CXCL8* and *CTLA4* were identified as significantly upmodulated genes in melanoma cultures. Investigation of circulating SHH levels revealed a modest increase in melanoma patients’ blood. This may be attributable to sample heterogeneity and limited cohort size. Further studies are underway in our laboratory to evaluate this parameter’s clinical value in more homogeneous groups stratified by stage, genotype, histological type, etc.

Treatment with sonidegib ameliorates several aspects of immunosuppressive features in melanoma cells. Particularly, the suppression of monocyte chemoattractant proteins is of interest due to the role of these proteins in orchestrating the recruitment of pro-tumorigenic M2 macrophages and tumor vascularization [91]. Drawing from cancer research data, particularly from melanoma studies, stimulation of IL-4 and RANTES appears to support the efficacy of therapies by modulating immune cell dynamics and inflammatory responses in the tumor microenvironment [69,92].

The most relevant effect of Hh inhibition by SMO inhibitors consists of the dose-dependent reduced proliferation of melanoma cells, which culminates in apoptosis at higher concentrations. However, taladegib barely impacted the proliferation rate of melanoma cells. Differences also emerged comparing vismodegib and sonidegib. According to these data, we previously demonstrated that despite the evidence of analogous clinical benefits, vismodegib and sonidegib have significantly different intensities of biological effect in vitro [49]. Due to a major grade of lipophilicity, sonidegib exhibits markedly higher plasma membrane permeability, achieving cytoplasmic concentrations approximately 20 times greater than those of vismodegib [49]. Consistent with observations reported for other SMO antagonists such as SANT1 and SANT2, sonidegib may inhibit the intracellular translocation of SMO to the primary cilium, thereby decreasing the amount of active SMO within the cell. Even when tested on melanoma cells, sonidegib was found to have a stronger cytotoxicity than vismodegib. Our results suggest a time-dependent sequence of drug effects involving S-phase arrest, likely due to impaired replication, as assessed by cell cycle analysis. Thereafter, the drug’s effect continues, and the cells begin to die, as reflected by the progressive reduction in the number of adherent cells and increased apoptosis.

Furthermore, our data are in line with other studies showing that inhibiting the Hh pathway reduces the migration of cancer cells by modifying cadherin-mediated adhesion properties, suggesting a favorable inhibitory effect on epithelial-to-mesenchymal transition. Consistent with our findings, Gunarta and collaborators reported that suppression of GLI1 decreases the invasive capacity of melanoma cells [93].

The ability of Hh inhibitors to promote E-cadherin upregulation and concurrent N-cadherin downregulation has been demonstrated in certain tumor cell types [50,94]. Nevertheless, as in our findings, it has also been reported that the modulation of E-cadherin in response to blocking the pathway does not accompany a parallel decrease in N-cadherin, suggesting that the EMT process is subjected to selective regulation [95]. Another group reported a direct correlation between the expression of GLI2, the release of metalloproteinases and the propensity to form melanoma bone metastasis in mice, as well as an inverse correlation with E-cadherin expression [96]. In line with this observation, we found a positive correlation between GLI2 and N-cadherin mRNA, but in this set of cell cultures no relation emerged matching the amount of E-cadherin and GLI2 transcripts.

The increased release of SHH following SMO inhibition suggests a finely tuned regulatory mechanism within this signaling pathway, underscoring its significance in melanoma biology. Confirming a ligand-dependent mechanism for abnormal Hh activation in melanoma cells, the circulating level of SHH was tendentially higher in patients compared to normal individuals. Together, these findings support the potential for translating this and previous research into clinical applications. As with other precision molecular therapies, blocking SMO carries the risk of resistance due to pre-existing or acquired mutations. In fact, despite significant successes in treating advanced and metastatic BCC, many patients develop clinical resistance during therapy, with about half of these harboring mutated *SMO* [97,98]. However, analyzing our institutional database containing NGS data from 1878 melanoma specimens, we found only three *SMO* mutations corresponding to about 0.16% of patients (manuscript in preparation), suggesting that genetic alterations in this gene are rare in untreated patients.

In conclusion, our findings demonstrate chronic Hh activation in human melanoma cells driven primarily by a ligand-dependent autocrine mechanism. Pharmacological modulation of Hh impacts the major critical features of cancer cells: the proliferation, the migration, and the crosstalk with components of proximal stroma, supporting the use of SMO inhibitors such as sonidegib and vismodegib for melanoma therapy. To open new therapeutic avenues, it will be crucial to collect more clinical data regarding Hh’s specific role in melanomagenesis and to perform clinical trials supporting the use of this type of drug. In line with this, very recently, a clinical investigation was conducted concerning the combination therapy of sonidegib with pembrolizumab (immunotherapy) for treating solid tumors including melanoma (https://www.clinicaltrials.gov/study/NCT04007744 accessed at the date of 29 December 2025).

## 4. Materials and Methods

Ethical statement. The study was conducted according to the guidelines of the Declaration of Helsinki and was approved by the Ethics Committee of IFO (Istituti Regina Elena e San Gallicano), protocol N. 1804/22 approved in date 13 December 2022.

Cell cultures. Melanoma samples were collected from patients at the San Gallicano Dermatological Institute, Istituti Fisioterapici Ospitalieri (IFO). Cells were isolated from surplus biopsy portions taken for histological analysis, ensuring that standard diagnostic procedures remained intact. Tissue was minced into small fragments and incubated with 0.35% collagenase for 45 min at 37 °C. After incubation, the mixture was centrifuged, resuspended, and cultured in OptiMEM medium (Life Technologies, Invitrogen, Milan, Italy) supplemented with 10% fetal bovine serum (FBS) and antibiotics (Euroclone S.p.A., Milan, Italy). All experiments used low-passage cell cultures (2–12). Patient’s characteristics are reported in Table S1. Normal human melanocytes were extracted from skin samples obtained from healthy volunteers subjected to plastic surgery. In this case, samples were selected to ensure sex and age matching with melanoma patients. Demographic details are summarized in Table S2.

MTT assay. Briefly, 2.0 × 10^4^ cells were seeded in 24-well plates for 24 h to adhere. Growth medium was then replaced with fresh medium containing treatments or vehicle at appropriate concentrations. After 24, 48 and 72 h, cells were incubated with 3-(4,5-dimethylthiazol)-2,5-diphenyl tetrazolium bromide (MTT) (Sigma-Aldrich, Merck, Milan, Italy) for 2 h. The medium was removed, and the resulting crystals were dissolved in DMSO. Absorbance was measured at 570 nm with 690 nm as a reference. Blank values were subtracted, and results expressed as a percentage of control absorbance. Experiments were performed in duplicates.

Cell counts by flow cytometry. At the experimental endpoint, single-cell suspensions were prepared and resuspended in 500 μL of PBS (Euroclone) containing 0.1% FBS prior to analysis. For each sample, 100 μL was acquired in duplicate. Flow cytometry was conducted using a Miltenyi MACSQuant Analyzer 10 (Miltenyi Biotec, Bologna, Italy). Experiments were performed in duplicates.

Immunofluorescence analysis. Melanoma cells were fixed with 4% paraformaldehyde, followed by 0.1% Triton-X 100 to allow permeabilization. Cells were incubated with the primary antibodies listed below: rabbit polyclonal anti-Ki67 antibody (1:300; Abcam, Cambridge Science Park, Cambridge, UK), rabbit monoclonal anti-E-cadherin antibody (1:100; antibodies.com), rabbit polyclonal N-cadherin antibody (1:400; Life Technologies, Invitrogen), rabbit polyclonal anti-SUFU antibody (1:150; Life Technologies, Invitrogen), mouse monoclonal anti-PTCH1 antibody (1:100; Origene Technologies, Rockville, MD, USA), mouse monoclonal anti-MITF antibody (Santa Cruz Biotechnology, Santa Cruz, CA, USA). The visualization of the primary antibodies was then achieved using goat anti-rabbit or anti-mouse Alexa Fluor 555 conjugates (Cell Signaling Technology, Danvers, MA, USA). Coverslips were mounted using ProLong Gold antifade reagent with DAPI (Life Technologies Invitrogen, Corporation, Eugene, OR, USA). Fluorescence signals were analyzed by recording stained images using a charge-coupled device camera (Zeiss, Oberkochen, Germany). The percentage of Ki67-positive cells was measured in a total of 11 melanoma cell lines. Results are expressed as the percentage of positive cells/total cells. Quantitative analysis of E-cadherin (*n* = 11) and N-cadherin (*n* = 9) fluorescence intensity was conducted using Zen 2.6 (Blue Edition) software (Zeiss).

Cell cycle distribution analysis. Melanoma cells were plated in 6 cm dishes with OptiMEM medium supplemented with 10% FBS and antibiotics. After 24 h, cells were synchronized by replacing the medium with OptiMEM containing 0.1% FBS and incubated for another 24 h. Then, cells were treated with sonidegib at 10 µM and 20 µM or DMSO as a control were included in full medium containing FBS, for 24, 48, and 72 h. At each time point, cells were harvested using 1X Trypsin-EDTA in PBS and fixed in ice-cold 70% ethanol for 20 min. After washing with 1X PBS, DNA staining was performed by incubating cells overnight at 4 °C in a buffer containing 1X PBS, propidium iodide (PI, 100 µg/mL), and RNase A (90 µg/mL).

A total of 10^4^ cells per sample were analyzed by flow cytometry (MACSQUANT X, Miltenyi Biotec) at a low flow rate. PI fluorescence was detected in the FL-3 channel on a linear scale using a 488 nm argon laser. Doublets and aggregates were excluded based on Area vs. Height bivariate plots of PI fluorescence in the same channel.

Cell cycle distribution was analyzed with FlowJo v10 (Becton Dickinson, BD Italia, Milan, Italy) using the Watson Pragmatic algorithm to estimate DNA content and calculate the percentage of cells in G1/G0, S, and G2/M phases. Identification of apoptotic cells by Annexin V/PI staining. Cell death and apoptosis were measured using the annexin V-FITC/propidium iodide (PI) double staining method after 48 h of treatment. At the endpoint, cells were harvested by trypsinization and suspended in staining buffer (10 mM HEPES/NaOH, pH 7.4, 140 mM NaCl, 2.5 mM CaCl_2_). The cells were then stained with FITC-labeled annexin V for 15 min at room temperature in the dark. Subsequently, samples were washed with PBS, centrifuged, and resuspended in PBS containing PI before being kept on ice until analysis. Flow cytometric analysis was performed using the MACSQuant Analyzer 10 (Miltenyi Biotec) with channels FL1 and FL3. Unstained cells served as negative controls. The percentages of single-positive cells (annexin V positive, indicating early apoptosis) and double-positive cells (annexin V plus PI positive, indicating late apoptosis or necrosis) are presented in the histograms.

Scratch Assay. Cells were seeded on 35 mm Petri dishes. At confluence, the cell monolayer was wounded with a pipette tip to obtain a standardized uniform cell-free area. After washing, cells were immediately fixed (time zero, T0) or treated with sonidegib or vehicle alone for 24 and 48 h. Samples were then fixed, and images were captured using a CCD camera (Zeiss). Migration was assessed in 11 melanoma cell lines. The distance between the edges of the scratched area of each sample was measured using Zen 2.6 software (Zeiss).

Detection of intracellular ROS levels. Production of ROS was assessed using the cell-permeable fluorescent dye 2′7′-dichlorodihydrofluorescein diacetate (H2DCF-DA, Sigma-Aldrich, Merck). Cells were incubated with 2.5 μmol L−1 H2DCF-DA for 30 min at 37 °C and 5% CO_2_ in phenol red-free, serum-starved medium, protected from light. After removing the probe solution, cells were washed with PBS, trypsinized, centrifuged at 1000 rpm, and resuspended in PBS. The oxidation of H2DCF into the fluorescent compound DCF by ROS was then quantified by flow cytometry using a Miltenyi MACSQuant Analyzer 10.

RT-PCR and gene expression array cards analysis. Total RNA was extracted using the Aurum Total RNA Mini Kit (Bio-Rad, Milan, Italy). Complementary DNA (cDNA) was synthesized from 1 µg of total RNA using the FirstAid cDNA Synthesis Kit (Fermentas, Thermo Fisher Scientific, Waltham, MA, USA). The cDNA samples were then loaded onto 384-well microfluidic cards pre-configured with selected primers and probe sets for the analysis of 93 target genes (Table S1) and three housekeeping genes (18S rRNA, glyceraldehyde-3-phosphate dehydrogenase (GAPDH), and β-actin). Real-time quantitative PCR was performed using the Applied Biosystems^®^ QuantStudio™ 7 Flex system (Thermo Fisher Scientific, Ferentino, FR, Italy). Samples were grouped into biogroups corresponding to homogeneous experimental conditions. Data analysis was conducted using a cloud-based platform using the global normalization method, employing one-way ANOVA with thresholds set at fold changes >2.0 or <0.5 and *p*-values ≤ 0.05.

Western blot analysis. Cell extracts were prepared using RIPA buffer supplemented with protease and phosphatase inhibitors. Proteins were separated by SDS–polyacrylamide gel electrophoresis and transferred onto nitrocellulose membranes. Membranes were blocked for 10 min at room temperature with EveryBlot Blocking Buffer (BioRad Laboratories, Milan, Italy) and subsequently cropped, guided by molecular weight markers, to enable the detection of different targets with distinct molecular weights before incubation with the appropriate primary antibodies: GLI2 (1:1000) (Cohesion Biosciences, Newport, London, UK), VEGF-A (1:1000) (Cell Signaling Technology, Danvers, MA, USA), E-Cadherin (1:1000) (antibodies.com), PRAME (1:1000) (Life Technologies Invitrogen, Eugene, OR, USA), GLI3 (1:1000) (R&D Systems, Minneapolis, MN, USA), N-Cadherin (1:1000) (Life Technologies Invitrogen), GADD45 and pγH2AX (1:1000) (Cell Signaling Technology), β-Actin (1:10,000) (Sigma Aldrich, Merck) and Cofilin (1:10,000) (BioRad Laboratories), which were used as a loading controls. After primary antibody incubation, membranes were incubated with the corresponding HRP-conjugated secondary antibodies: anti-mouse and anti-rabbit (Cell Signaling Technology), and anti-goat (Santa Cruz Biotechnology Inc., Dallas, TX, USA). Image acquisition and densitometric analysis were performed using the UVITEC Mini HD9 system (Alliance UVItec Ltd., Cambridge, UK) in at least six independent cell lines. The PM2610 ExcelBand™ Enhanced 3-color High Range Protein Marker (SMOBIO Technology, Inc., Hsinchu City, Taiwan) was used for monitoring protein separation during SDS-polyacrylamide gel electrophoresis.

Protein Arrays. The expression of 58 human cytokines, immune system mediators and 40 growth factors was analyzed using a commercially available antibody array system (RayBio^®^QAH-CYT-1, QAH-CHE-1, QAH-INF-1, QAH-GF-1, Inc., Peachtree Corners, GA, USA), according to the manufacturer’s instructions. Cells were seeded in 10 cm culture dishes and treated (or not) with sonidegib 10 and 20 μM for 48 h before conditioned medium collection. Slides were incubated with 100 μL of 1× blocking buffer for 30 min at room temperature (RT), then incubated overnight at 4 °C with 100 μL of conditioned medium or standards. Afterwards, slides were washed three times with 100 μL of 1× wash buffer I and twice with 100 μL of 1× wash buffer II at RT. Then the slides were incubated with biotin-conjugated primary antibodies for 2 h at RT, followed by the same washing steps before incubation with 1:1000 diluted horseradish peroxidase-conjugated streptavidin for 1 h. All the slides were stored at 4 °C in was buffer for a week before being sent to Ray Biotech for scanning, data extraction and analysis. Obtained data were normalized using the total protein amount of each experimental endpoint.

DNA extraction and Next Generation Sequencing (NGS) Analysis. Genomic DNA was extracted using the DNeasy Blood and Tissue Kit (Qiagen Italia, Milan, Italy) and quantified with a Qubit Fluorometer (Thermo Fisher Scientific) using the Qubit^®^ dsDNA HS Assay Kit. A custom next-generation sequencing (NGS) panel (Illumina Inc., San Diego, CA, USA) was designed to target seven key genes involved in the Hh signaling pathway: *SHH*, *DHH*, *IHH, GLI1*, *GLI2*, *GLI3*, *PTCH1*, *PTCH2*, *SMO*, and *SUFU*. Library preparation was performed using 10 ng of DNA with the AmpliSeq Library Plus for Illumina kit (Illumina). Each library was uniquely barcoded with the AmpliSeq™ CD Index Set A (Illumina), diluted to a final concentration of 100 pM, and pooled in equimolar amounts. Sequencing was carried out on the MiSeqDX™ platform (Illumina) utilizing the MiSeq v2 Reagent Kit. For data analysis, sequencing reads were processed with nf-core/sarek v3.4.3: alignment to GRCh37 was performed with BWA-MEM, duplicate marking applied, inline UMIs extracted with UMI-tools, and analysis restricted to the custom panel BED regions [82]. Somatic SNVs and indels were called with Mutect2 and Strelka2.

All cell lines were genotyped for *BRAF* (exon 11 and exon 15) and *NRAS* (exon 2, exon 3 and exon 4) hotspot regions via targeted NGS. In this case, genomic DNA was analyzed using the 50-gene Ion AmpliSeq™ Cancer Hotspot Panel v2 (Thermo Fisher Scientific), according to the manufacturer’s protocols.

Enzyme-linked Immunosorbent Assay (ELISA) for SHH quantification. SHH concentration was measured using a human SHH ELISA kit (Abbexa Ltd., Cambridge, UK, cat. no: abx253573) following the manufacturer’s instructions. Absorbance was read at 450 nm with a microplate reader (DTX 880 Multimode Detector, Beckman Coulter, Milan, Italy). In the case of supernatants and normalized to total protein content. Assays were performed in duplicate.

Statistical analysis. Statistical analyses were performed using GraphPad Prism (version 10.3.0; La Jolla, CA, USA). Data normality was evaluated using the Shapiro–Wilk test. SHH measurements in serum and supernatants were not normally distributed and were therefore analyzed using the Mann–Whitney U test for unpaired comparisons and the Wilcoxon signed-rank test for paired data, respectively. For grouped data related to cell-cycle analysis, a two-way ANOVA was conducted, followed by Tukey’s multiple-comparison test. Correlation was evaluated by the coefficient of Pearson’s test (r).

All the other quantitative data were reported as mean ± standard deviation (SD). The number of biological replicates is indicated in the figure legends. In this case, student t-test was used to assess statistical significance. * *p* ≤ 0.05, ** *p* ≤ 0.01, *** *p* ≤ 0.005 and **** *p* ≤ 0.005.

## Figures and Tables

**Table 1:** Inflammation. Fold-change modification of inflammatory factors released by melanoma cell treated with sonidegib compared to untreated samples. * *p* < 0.05; ** *p* < 0.01; **** *p* < 0.001.

Target	Son 20 μM
**Eotaxin**	140.9 ± 278.1
**GCP-2**	1.399 ± 0.3418 **
**IL-1a**	4.841 ± 10.74
**IL-1b**	8.799 ± 19.28
**IL-4**	**1.985** ± 1.271 *
**IL-5**	32.04 ± 88.84
**IL-9**	5.170 ± 5.882
**IL-10**	5.785 ± 14.69
**LIGHT**	0.2846 ± 0.3462 **
**Lymphotactin**	6.578 ± 12.90
**MCP-2**	0.00 ± 0.00 ****
**MCP-3**	0.1959 ± 0.3025 **
**MCP-4**	0.00 ± 0.00 ****
**MDC**	0.00 ± 0.00 ****
**MIF**	4.622 ± 6.988
**MIP-1a**	2.397 ± 2.674
**MIP-1b**	4.976 ± 12.15
**MSP**	0.00 ± 0.00 ****
**RANTES**	10.74 ± 35.76
**SDF-1a**	1.381 ± 0.4057 *
**TARC**	0.1795 ± 0.2539
**TNFa**	2.963 ± 3.502
**TSLP**	1.310 ± 0.3089 *

**Table 2:** Growth factors. Fold-change modification of growth factors released by melanoma cell treated with sonidegib compared to untreated samples. * *p* < 0.05; ** *p* < 0.01.

Target	Son 20 μM
**GDF-15**	1.473 ± 0.5353 **
**Insulin**	1.781 ± 1.169 *
**OPG**	1.314 ± 0.3189 **

## Data Availability

The data presented in this study are available on request from the corresponding author (B.B.). The data are not publicly available to protect study participant privacy.

## Supplementary Materials

The following supporting information can be downloaded at https://www.mdpi.com/article/10.3390/ijms27020762/s1.
