# Supplementary material for: Aberrant Activation of the Hedgehog Pathway in Cutaneous Melanoma: Therapeutic Potential of Pharmacological Inhibitors
Source: Int J Mol Sci. 2026 Jan 12;27(2):762. doi: 10.3390/ijms27020762 (PMC12840925; doi:10.3390/ijms27020762)
Supplement: Supplementary file 1 [file ijms-27-00762-s001.zip › ijms-4050687-supplementary.pdf]

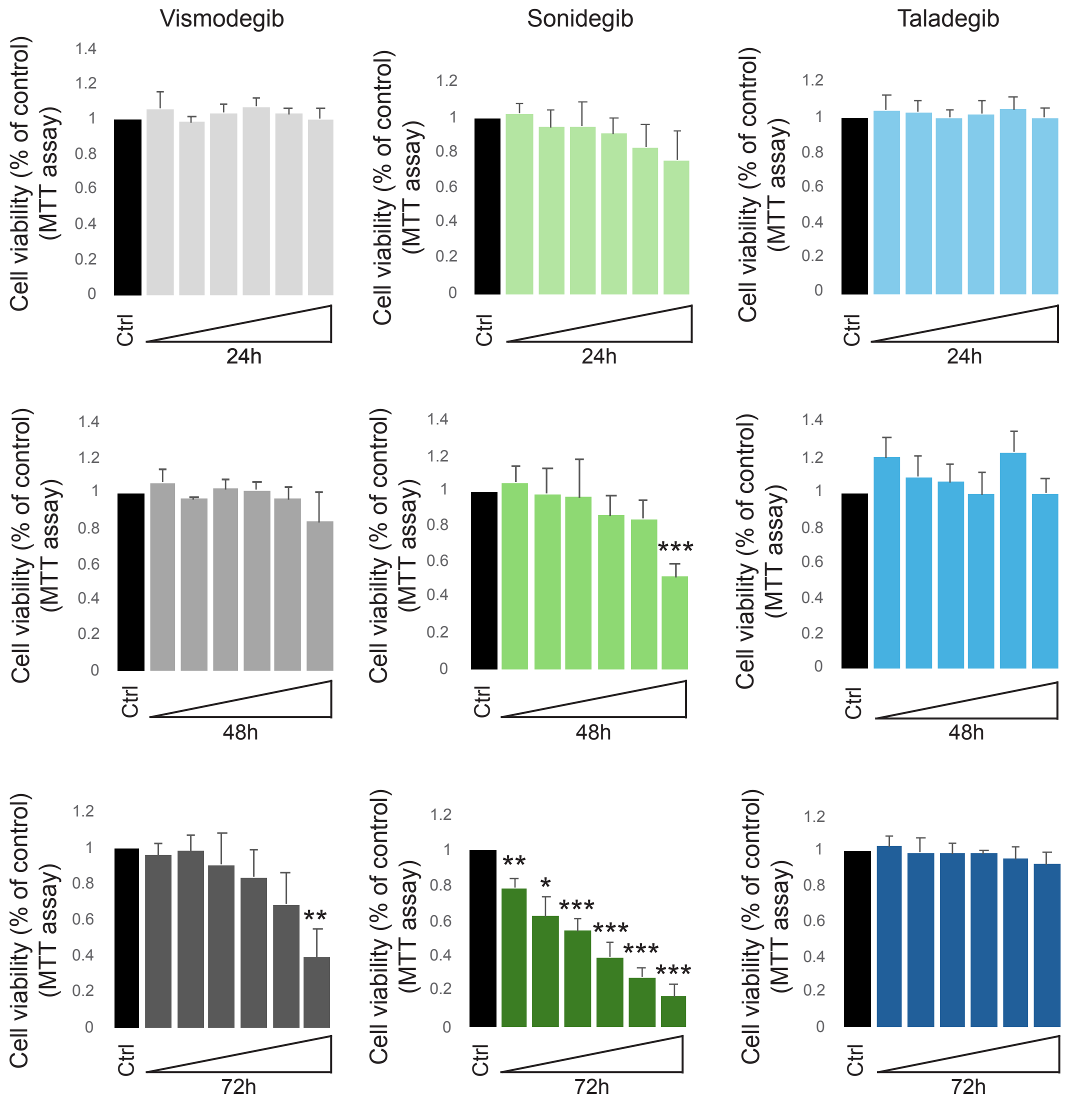

Supplementary Figure S1. Time and dose-dependent effect of Hh inhibitors evaluated by MTT assay. Three melanoma cell lines were exposed to vismodegib, sonidegib and taladegib in duplicate. *p<0.05; ** p<0.01; ***p<0.005.

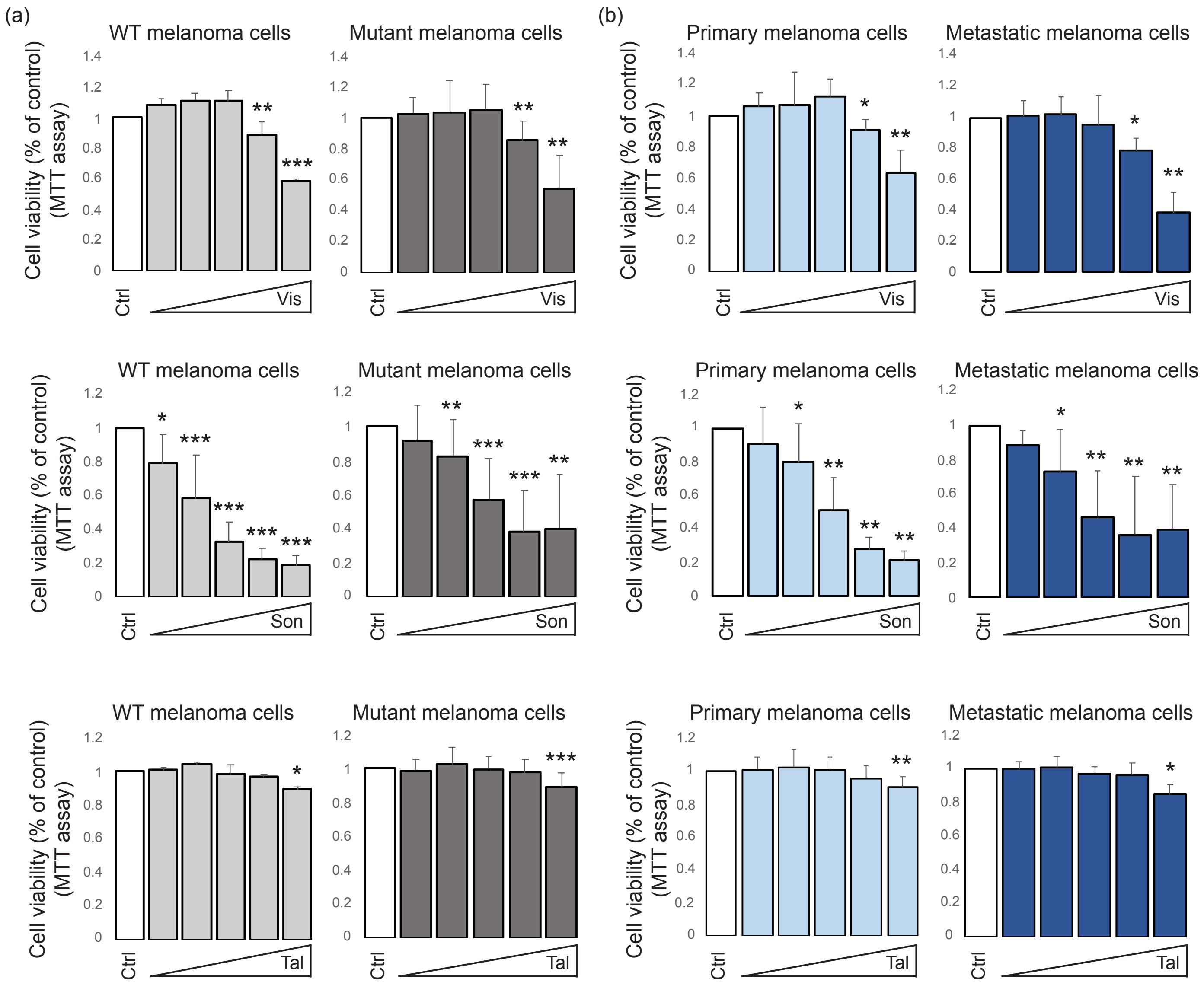

Supplementary Figure S2. (a) Comparative analysis of the cytotoxic effect of vismodegib, sonidegib and taladegib on wild-type (n=5) and BRAF/NRAS mutated cells (n=13). (b) Comparative analysis of the cytotoxic effect of vismodegib, sonidegib and taladegib (72 hours) on primary (n=8) and metastatic (n=5) cell cultures. Experiments were performed in duplicate. Statistical analysis did not evidence differences between the two melanoma types. *p<0.05; ** p<0.01; ***p<0.005.

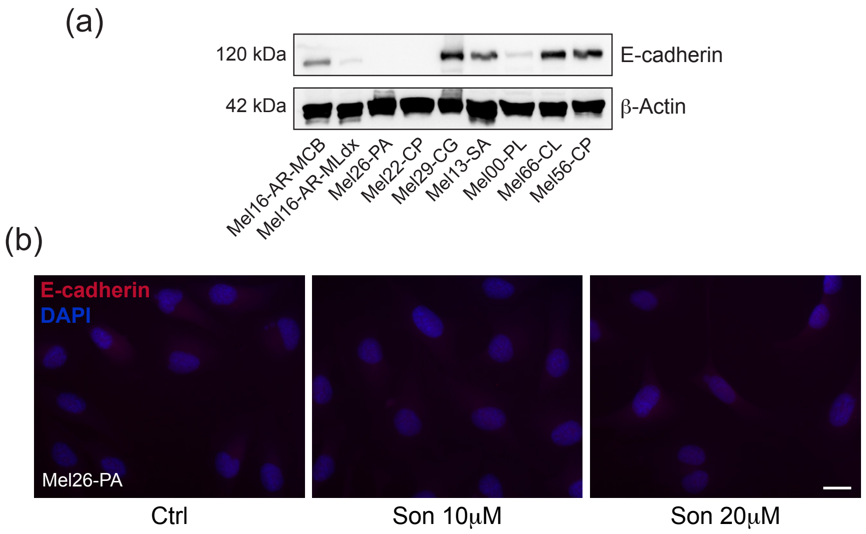

Supplementary Figure S3. (a) Western blot analysis of E-cadherin expression in a panel of melanoma cell lines. Data evidence a high heterogeneity in the amount of this adhesion protein that completely loss in some samples (b) Representative immunofluorescence analysis of a cell lines characterized by undetectable expression level of E-cadherin. In this case the treatment with 10 and 20 μM Son for 48 hours failed to re-induce the protein. Nuclei are counterstained with DAPI. Scale bar: 20 μm.

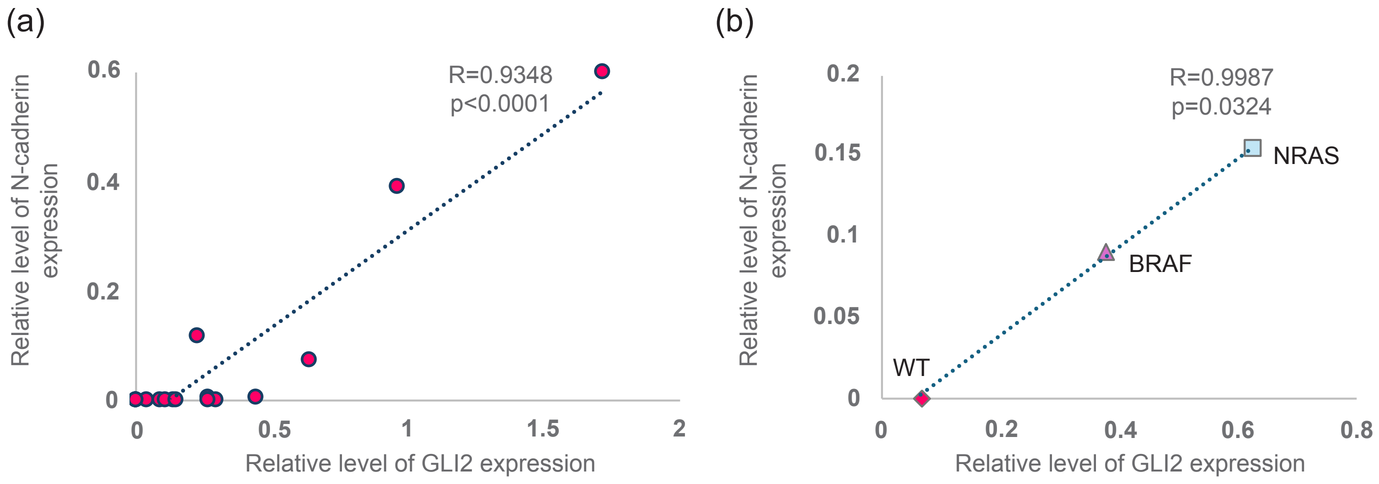

Supplementary Figure S4. Correlation of GLI2 and N-cadherin level of expression. (a) The value obtained by RT-PCR for the expression level of GLI2 and N-cadherin mRNA of single melanoma cell lines (n=19) were used to test the correlation, and data demonstrated a direct correlation between these mRNAs. (b) The analysis was repeated grouping samples by the mutation of BRAF (n=8) NRAS (n=3) genes or wild-type configuration for both genes (n=6). Data confirmed the correlation between N-cadherin and GLI2 mRNA level and further evidenced the highest level for both proteins in cells carrying mutated NRAS.

Table S1. Patient's characteristics

Tumor Cell Lines	Sex/Age	Primary/ Metastatic	TNM	Stage	BRAF Exon15	NRAS Exon3
Mel8-SM	52/M	Primary	pT2bN2M1a	IV	WT	Q61K
Mel13-SA	62/M	Metastatic (Cutaneous)	pT3bN0M1	IV	WT	Q61R
Mel16-AR-P	46/F	Primary	pT4bN3M1	IVB	WT	WT
Mel16-AR-MCA	46/F	Metastatic (Cutaneous)	pT4bN3M1	IVB	WT	WT
Mel16-AR-MCB	46/F	Metastatic (Cutaneous)	pT4bN3M1	IVB	WT	WT
Mel16-AR-MLsx	46/F	Metastatic (Lymph node)	pT4bN3M1	IVB	WT	WT
Mel16-AR-MLdx	46/F	Metastatic (Lymph node)	pT4bN3M1	IVB	WT	WT
Mel26-PA	45/F	Primary	pT1bN0M0	IB	V600E	WT
Mel29O-CG	38/M	Primary	pT3bN3M0	IIIC	V600E	WT
Mel29T-CG	38/M	Primary	pT3bN3M0	IIIC	V600E	WT
Mel35-TE	65/F	Primary	pT4bN0M0	IIB	WT	Q61R
Mel56-CP	48/M	Primary	pT4bN0M0	IIC	V600R	WT
Mel60-BM	83/F	Primary	pT4bNxMx	IIID	V600E/V600K	WT
Mel66-CL	44/M	Primary	pT4bN0M0	IIC	V600E/V600E	WT
Mel67-NA	69/M	Primary	pT1bN2M0	IIIA	V600E/V600K	WT
Mel00-PL	69/M	Metastatic (Cutaneous)	pT4bN3M1	IVB	V600E	WT
Mel73-MA	44/M	Primary	pT3bN0M0	IIIC	WT	WT
Mel77-DM-P	71/M	Primary	pT4bN3M0	IIID	V600E	WT
Mel77-DM-MLdx	71/M	Metastatic (Lymph node)	pT4bN3M0	IIID	V600E	WT
Mel22-MC	67/F	Primary	pT4bN3M0	IIIB	V600E	WT
Mel68-DBG#	74/M	Primary	pT4bN0M0	IVB	V600K	WT
Mel84-MC#	79/F	Primary	pT4bN0M0	IVB	V600E	WT
Mel85-AG#	89/F	Primary	pT4bNxMx	IVB	WT	Q61R

Table S2. Healthy donor's characteristics

Melanocyte Cell Lines	Sex/Age	Body Area
NHMA20	39/M	Abdomen
NHMA38	62/M	Abdomen
NHMBr13	47/F	Arm
NHMD24	40/M	Dorsal
NHMD30	33/F	Dorsal
NHMF5	70/M	Flank
NHMG22	53/F	Leg
NHMRa8	19/M	Behind the ear
NHMS8	30/F	Breast
NHMS10	53/F	Breast
NHMS15	69/F	Breast
NHMS18	52/F	Breast
NHMS26	46/F	Breast
NHMSp3	44/M	Shoulder
NHMp111	89/M	Foreskin

cell line used only for NGS analysis

Table S3. List of analyzed genes and the corresponding primer sets used for TaqMan amplification

Primers reference	Gene name	Primers reference	Gene name
**A2M-Hs00929971_m1**	alpha-2-macroglobulin	**IGFBP6-Hs00181853_m1**	insulin like growth factor binding protein 6
**ACTA2-Hs00426835_g1**	actin, alpha 2, smooth muscle, aorta	**IGFBP7-Hs00266026_m1**	insulin like growth factor binding protein 7
**AXIN2-Hs00610344_m1**	axin 2	**IL18-Hs01038788_m1**	interleukin 18
**BAP1-Hs01109276_g1**	BRCA1 associated protein 1	**IL1A-Hs00174092_m1**	interleukin 1 alpha
**BCL2-Hs04986394_s1**	BCL2, apoptosis regulator	**IL1B-Hs01555410_m1**	interleukin 1 beta
**BCL6-Hs00153368_m1**	B-cell CLL/lymphoma 6	**IL6-Hs00174131_m1**	interleukin 6
**BDKRB1-Hs00664201_s1**	bradykinin receptor B1	**LEF1-Hs01547250_m1**	lymphoid enhancer binding factor 1
**BDKRB2-Hs00176121_m1**	bradykinin receptor B2	**MAPK14-Hs01051152_m1**	mitogen-activated protein kinase 14
**BIRC5-Hs04194392_s1**	baculoviral IAP repeat containing 5	**MAPK1-Hs01046830_m1**	mitogen-activated protein kinase 1
**BMP2-Hs00154192_m1**	bone morphogenetic protein 2	**MAPK3-Hs00385075_m1**	mitogen-activated protein kinase 3
**BMP4-Hs03676628_s1**	bone morphogenetic protein 4	**MAPK8-Hs01548508_m1**	mitogen-activated protein kinase 8
**CASP3-Hs00234387_m1**	caspase 3	**MC1R-Hs00267167_s1**	melanocortin 1 receptor
**CCL19-Hs00171149_m1**	C-C motif chemokine ligand 19	**MCL1-Hs06626047_g1**	BCL2 family apoptosis regulator
**CCL2-Hs00234140_m1**	C-C motif chemokine ligand 2	**MITF-Hs01117294_m1**	melanogenesis associated transcription factor
**CCNB1-Hs01030099_m1**	cyclin B1	**MMP1-Hs00899658_m1**	matrix metallopeptidase 1
**CD36-Hs00354519_m1**	CD36 molecule	**MMP2-Hs01548727_m1**	matrix metallopeptidase 2
**CD40-Hs01002915_g1**	CD40 molecule	**MMP9-Hs00957562_m1**	matrix metallopeptidase 9
**CDH1-Hs01023895_m1**	cadherin 1	**PAX3-Hs07288494_m1**	paired box 3
**CDH2-Hs00983056_m1**	cadherin 2	**PRAME-Hs01022301_m1**	preferentially expressed antigen in melanoma
**CDKN2A-Hs00923894_m1**	cyclin dependent kinase inhibitor 2A	**PTCH1-Hs00181117_m1**	patched 1
**CERS1-Hs04195319_s1**	ceramide synthase 1	**PTCH2-Hs00184804_m1**	patched 2
**CERS6-Hs00826756_m1**	ceramide synthase 6	**PTGS2-Hs00153133_m1**	prostaglandin-endoperoxide synthase 2
**CES1-Hs00275607_m1**	carboxylesterase 1	**SCD-Hs01682761_m1**	stearoyl-Co
**CSF1-Hs00174164_m1**	colony stimulating factor 1	**SFRP1-Hs00610060_m1**	secreted frizzled related protein 1
**CSF2-Hs00929873_m1**	colony stimulating factor 2	**SFRP2-Hs01564480_m1**	secreted frizzled related protein 2
**CTLA4-Hs00175480_m1**	cytotoxic T-lymphocyte associated protein 4	**SLC2A1-Hs00892681_m1**	solute carrier family 2 member 1
**CTNNB1-Hs00355045_m1**	catenin beta 1	**SLC2A4-Hs00168966_m1**	solute carrier family 2 member 4
**CXCL10-Hs00171042_m1**	C-X-C motif chemokine ligand 10	**SMAD7-Hs00998193_m1**	SMAD family member 7
**CXCL16-Hs00222859_m1**	C-X-C motif chemokine ligand 16	**SMO-Hs01090242_m1**	smoothened, frizzled class receptor
**CXCL8-Hs00174103_m1**	C-X-C motif chemokine ligand 8	**SOX10-Hs00366918_m1**	SRY-box 10
**DKK2-Hs00205294_m1**	dickkopf WNT signaling pathway inhibitor 2	**SOX2-Hs04234836_s1**	SRY-box 2
**DKK3-Hs00247429_m1**	dickkopf WNT signaling pathway inhibitor 3	**SOX9-Hs00165814_m1**	SRY-box 9
**EDN1-Hs00174961_m1**	endothelin 1	**SUFU-Hs00960520_m1**	SUFU negative regulator of hedgehog signaling
**EDN2-Hs01012711_g1**	endothelin 2	**TCF7-Hs01556515_m1**	transcription factor 7 (T-cell specific, HMG-box)
**FABP3-Hs07287862_m1**	fatty acid binding protein 3	**TGFB1-Hs00998133_m1**	transforming growth factor beta 1
**FAP-Hs00990791_m1**	fibroblast activation protein alpha	**TIMP2-Hs00234278_m1**	TIMP metallopeptidase inhibitor 2
**FASN-Hs01005622_m1**	fatty acid synthase	**TNF-Hs00174128_m1**	tumor necrosis factor
**FGF19-Hs00192780_m1**	fibroblast growth factor 19	**TYR-Hs00165976_m1**	tyrosinase
**FGF20-Hs00173929_m1**	fibroblast growth factor 20	**VCAM1-Hs01003372_m1**	vascular cell adhesion molecule 1
**GLI1-Hs00171790_m1**	GLI family zinc finger 1	**VEGFA-Hs00900055_m1**	vascular endothelial growth factor A
**GLI2-Hs01119974_m1**	GLI family zinc finger 2	**WIF1-Hs00183662_m1**	WNT inhibitory factor 1
**GLI3-Hs00609233_m1**	GLI family zinc finger 3	**WNT10B-Hs00928823_m1**	Wnt family member 10B
**HGF-Hs00300159_m1**	hepatocyte growth factor	**WNT1-Hs00180529_m1**	Wnt family member 1
**HHIP-Hs01011015_m1**	hedgehog interacting protein	**WNT2B-Hs00921615_m1**	Wnt family member 2B
**ICAM3-Hs00913466_g1**	intercellular adhesion molecule 3	**WNT3A-Hs00263977_m1**	Wnt family member 3A
**IGF1-Hs01547656_m1**	insulin like growth factor 1	**WNT5A-Hs00998537_m1**	Wnt family member 5A
**IGF1R-Hs00609566_m1**	insulin like growth factor 1 receptor		

Table S4. NGS panel description

Genes	Location	Locus
GLI1	NM_005269	chr12:57857368
GLI2	NM_005270	chr2:121554838
GLI3	NM_000168	chr7:42003871
PTCH1	NM_001083602	chr9:98209139
PTCH2	NM_003738	chr1:45286347
SMO	NM_005631	chr7:128828873
SUFU	NM_016169	chr10:104263779
SHH	NM_000193	chr7:155592639
IHH	NM_002181	chr2:219919873
DHH	NM_021044	chr12:49483582

Table S5 Inflammation. Fold-change modification of inflammatory factors released by melanoma cell treated with sonidegib compared to untreated samples.

Target	Son 20
6Ckine	0.8707±0.9296
Axl	1.490±1.474
BTC	0.5931±0.5825
CXCL16	1.104±0.9331
ENA78	1.396±0.4342
GM-CSF	1.984±2.550
GRO	0.5971±0.6714
HCC-4	1.492±0.9553
IL-2	0.55371±0.7830
IL-6	1.940±2.187
IL-8	1.940±1.892
IL-13	0.6164±0.5369
IL-17F	1.408±0.4781
IL-28A	1.912±3.725
IL-18BPa	1.373±1.591
IL-29	1.313±0.3652
INTg	0.9931±0,9169
IP-10	0.5826±0.9125
LIF	0.8358±0.8801
MCP-1	0.8828±0.7074
MIP-3a	1.375±0.5095
MPIF-1	1.711±2.428
NAP-2	1.471±0.9671
OPN	1.420±1.625
PARC	1.143±1.057
PF4	1.292±1.648
TECK	0.6218±0.7085

Table S6 Growth factors. Fold-change modification of growth factors released by melanoma cell treated with sonidegib compared to untreated samples.

Target	Son 20
BMP-4	1.697±2.434
EGF	0.5267±0.5326
EGFR	1.410±0.6229
GH	1.803±1.906
HB-EGF	0.9508±1.014
IGFBP-2	1.274±1.552
IGFBP-6	0.9131±0.7966
MCSF-R	1.051±0.06341
NGF-R	1.376±0.7787
PDGF-A	1.418±1.895
PIGF	1.106±0.06839
SCF-R	1.585±1.179
TGF-a	1.087±0.2695
TGF-b1	0.5417±0.7940
VEGF	0.9042±0.5535
VEGF-D	0.6206±0.9538
